# In vivo self-assembled nano-PROTAC for the dual degradation of AR and HSP90 to overcome castration-resistant prostate cancer resistance

**DOI:** 10.1038/s41392-025-02444-z

**Published:** 2025-10-15

**Authors:** Fei-Ya Yang, Ni-Yuan Zhang, Yang Yang, Dong Chen, Li-Yuan Wu, Wen-Kuan Wang, Hao-Xi Wang, Zhuan Wen, Ming-Ze Cai, Hao-Ze Li, Haojie Huang, Hong-Wei An, Hao Wang, Nian-Zeng Xing

**Affiliations:** 1 Department of Urology, National Cancer Center/National Clinical Research Center for Cancer/Cancer Hospital, Chinese Academy of Medical Sciences and Peking Union Medical College, Beijing, China; 2 State Key Laboratory of Molecular Oncology, National Cancer Center/National Clinical Research Center for Cancer/Cancer Hospital, Chinese Academy of Medical Sciences and Peking Union Medical College, Beijing, China; 3 Beijing Key Laboratory of Urologic Cancer Cell and Gene Therapy, National Cancer Center/National Clinical Research Center for Cancer/Cancer Hospital, Chinese Academy of Medical Sciences and Peking Union Medical College, Beijing, China; 4 CAS Center for Excellence in Nanoscience, CAS Key Laboratory for Biomedical Effects of Nanomaterials and Nanosafety, National Center for Nanoscience and Technology (NCNST), Zhongguancun, Beijing, China; 5 Henan Institute of Advanced Technology, Zhengzhou University, Zhengzhou, China; 6 Departments of Pathology, State Key Laboratory of Molecular Oncology, National Cancer Center/National Clinical Research Center for Cancer/Cancer Hospital, Chinese Academy of Medical Sciences and Peking Union Medical College, Beijing, China; 7 Department of Urology, The First Affiliated Hospital, Institute of Urologic Science and Technology, The First Affiliated Hospital, Zhejiang University School of Medicine, Hangzhou, China; 8 Center of Materials Science and Optoelectronics Engineering, University of Chinese Academy of Sciences, Beijing, China

**Keywords:** Nanobiotechnology, Drug delivery

## Abstract

Castration-resistant prostate cancer demonstrates intrinsic or acquired resistance to second-generation androgen-targeted therapies, posing a challenge in clinical treatment. In this study, on the basis of in vivo self-assembly nanotechnology, we designed a PSMA-targeted nano-PROTAC with a proximity degradation effect. Nano-PROTAC not only precisely degrades the AR receptor but also cleverly degrades the HSP90 that is closely bound to the AR receptor, utilizing the spatial distance self-adaptive characteristics of its nanostructure. In the 22Rv1 cell model, Nano-PROTAC degraded 80% of the AR protein and 65% of the HSP90 protein. More importantly, nano-PROTAC could degrade 74% of the AR splice variant AR-V7 protein, showing the potential ability to overcome drug resistance. We further constructed an enzalutamide-resistant xenograft tumor mouse model to evaluate the therapeutic effect of the Nano-PROTAC. Compared with the combination treatment group of AR and HSP90 inhibitors (enzalutamide and pimitespib), the nano-PROTAC treatment group presented a high tumor growth inhibition value of up to 78% and a median survival extension of 15 days. Nano-PROTACs that simultaneously degrade AR and HSP90 can overcome the resistance of prostate cancer to PSMA- and AR-positive castration-resistant prostate cancer, except for neuroendocrine prostate cancer, which provides a new therapeutic strategy for the treatment of prostate cancer.

## Introduction

Prostate cancer is the second most common cancer in men, with an estimated 1.46 million diagnoses in 2022.^1^ Approximately 10–20% of advanced prostate cancers develop into castration-resistant prostate cancer (CRPC) within 5 years, and at least 84% of CRPC patients have metastasized cancer at the time of diagnosis.^2,3^ The prognosis for patients with advanced prostate cancer is poor, with a 5-year survival rate that remains very low. Prostate cancer cells rely on the activity of the transcription factor androgen receptor (AR), which is a primary therapeutic target for both primary and advanced diseases.^4^ Although treatments based on second-generation androgen receptor-targeting agents (including enzalutamide and abiraterone) have significantly improved patient progression-free survival and overall median survival rates, the 5-year survival rate for advanced prostate cancer patients is still less than 30%.^5^ Most metastatic patients, after 14–30 months of androgen deprivation therapy, inevitably develop CRPC, with an average survival period of no more than 2 years. Currently, CRPC is a challenge in clinical treatment and a hotspot in basic research, with a lack of effective methods. Compared with bicalutamide, enzalutamide, a new generation of selective AR antagonists, has a greater affinity for the AR. However, ~20–40% of prostate cancer patients are intrinsically resistant to enzalutamide, and even those who are initially sensitive to enzalutamide may develop resistance over time.^5^ One of the main reasons for this resistance is the presence of AR splice variants. AR-V7 is an AR splice variant. In the absence of androgen, AR-V7 can still induce ligand-independent transcription and can regulate gene expression.^6^ Therefore, it is considered to be the most relevant subtype among splice variants to CRPC.^5^ The resistance to potent second-generation antiandrogens, such as enzalutamide and abiraterone acetate, is attributed to the overexpression of AR-V7.^7^ Although some innovative therapies, such as PROTAC technology, can efficiently degrade AR proteins, their effects on AR-V7 are relatively weak. Therefore, how to address its resistance has become a focus in clinical medicine.

Heat shock protein 90 (HSP90) is a molecular chaperone that assists other proteins in folding into their natural conformations.^8^ It stabilizes client proteins against various stresses in cells and is a key regulator of protein balance. HSP90 clients include oncogenic proteins such as v-Src, Bcr-Abl, c-Met, and Plk1.^9,10^ Therefore, inhibiting HSP90 is considered a promising cancer treatment strategy. In prostate cancer, HSP90 folds the AR into the correct conformation in the cytoplasm to achieve stable, high-affinity ligand binding.^11^ When HSP90 function is inhibited, the AR protein misfolds due to the loss of chaperone protection and is eventually degraded by the ubiquitin‒proteasome system, thereby blocking the AR-dependent transcriptional program.^12,13^ The design strategy of traditional HSP90 inhibitors focuses mainly on competitive inhibition of the ATP binding pocket.^14,15^ Since this site is highly conserved across different species, such inhibitors (such as the first clinically introducedamycin derivative 17-AAG) can simultaneously trigger the degradation of more than 200 client proteins, including AR, HER2, Akt, BCR-ABL, and Raf-1.^16^ Although 17-AAG significantly inhibited the growth of prostate cancer cells in vitro,^17–19^ due to severe systemic toxicity and suboptimal pharmacokinetic properties, 17-AAG failed to achieve effective therapeutic concentrations in patients with metastatic CRPC,^20–22^ highlighting the limitations of traditional HSP90 inhibitors in clinical applications. Currently, novel AR antagonist-HSP90 inhibitor conjugates can degrade not only the full-length AR protein but also the AR splice variant AR-V7.^23^ As the latter serves as an important compensatory mechanism in the AR signaling pathway, its degradation is highly important for overcoming therapeutic resistance in CRPC.^8,24^

**Scheme 1:**
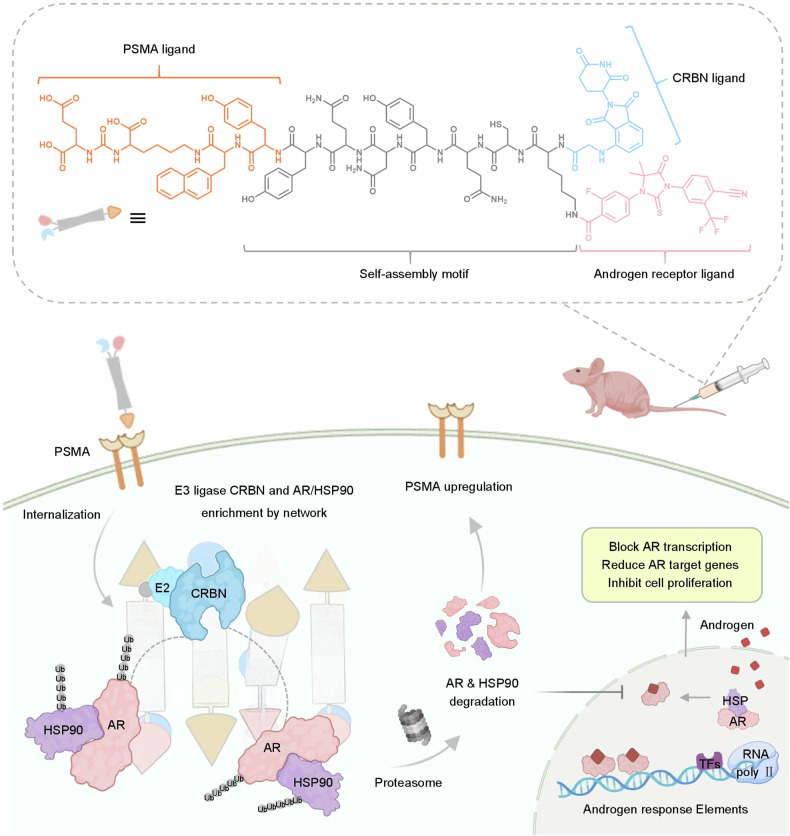
Schematic illustration of in vivo self-assembled nano-PROTAC for the dual degradation of AR and HSP90

In recent years, nanotechnology-based PROTACs have developed rapidly and demonstrated prominent advantages in overcoming the limitations of classical PROTACs.^25^ including poor solubility, low membrane permeability, and off-target effects.^25–27^ For example, by leveraging nanostructures,^28,29^ they enhance drug delivery,^30^ target specificity, and controlled release,^31^ thereby expanding the application of targeted protein degradation therapy. Additionally, the anthook effect potential of fibrous nanostructures further broadens the scope of targeted protein degradation therapy.^25,32^ Self-assembled nano-PROTACs form polynary complexes via multiple supramolecular interactions at high concentrations,^33^ directly contributing to this effect,^34^ a property distinguishing them from other nanoscale PROTACs,^35^ including SM-PROTAC, GNC-PROTAC, CDTAC, and DbTAC.^32,36^ Therefore, on the basis of the in vivo self-assembly nanotechnology developed by our group, we designed a PSMA-targeted nano-PROTAC (Psa-AR) with a proximity degradation effect.^32,37^ Psa-AR was engineered as a modular system comprising four functional domains: a PSMA-targeting ligand, a self-assembling peptide, an AR-binding moiety, and an E3 ligase ligand. Following PSMA-mediated tumor-specific cellular internalization, Psa-AR self-assembles into β-sheet nanofibrils within the cytoplasm (Scheme 1). When AR with HSP90 forms a complex state in the cytoplasm, Nano-PROTAC not only precisely degrades the AR receptor but also cleverly degrades the HSP90 that is closely bound to the AR receptor, utilizing the spatial distance self-adaptive characteristics of its nanostructure. In the 22Rv1 cell model, Nano-PROTAC degraded 80% of the AR protein and 65% of the HSP90 protein. More importantly, nano-PROTAC could degrade 74% of the AR splice variant AR-V7 protein, showing the potential ability to overcome drug resistance. We further constructed an enzalutamide-resistant xenograft tumor mouse model to evaluate the therapeutic effect of the Nano-PROTAC. Compared with the combination treatment group of AR and HSP-90 inhibitors (enzalutamide and pimitespib), the nano-PROTAC treatment group presented a high tumor growth inhibition (TGI) value of up to 78% and a median survival extension of 15 days. The design of the targeted recognition function and proximity degradation effect of the PSMA receptor further reduces the risk of off-target toxicity in PROTACs. Nano-PROTACs that simultaneously degrade AR and HSP90 can overcome the resistance of prostate cancer, except for neuroendocrine prostate cancer, to PSMA- and AR-positive CRPC,^38^ which provides a new therapeutic strategy for the treatment of prostate cancer.^39^

## Results

### Nano-PROTAC design and functional validation

**Fig. 1:**
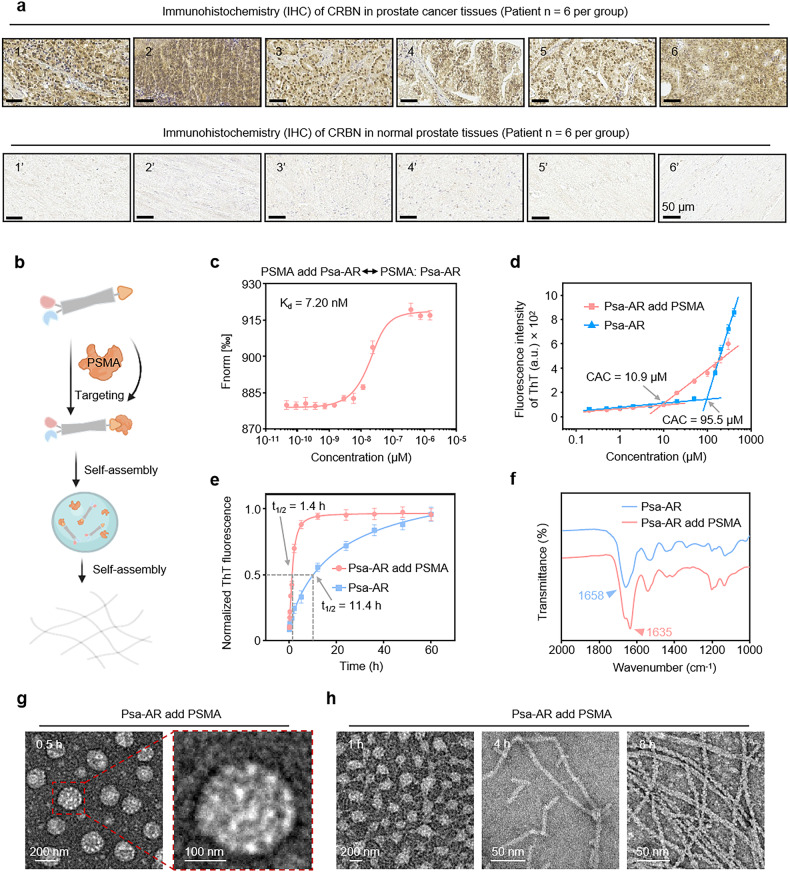
Protein-induced self-assembly behaviors of Psa-AR in vitro. **a** Six tissue samples from patients with prostate tumors (1–6) and six from patients with normal prostates (1’–6’) were obtained clinically. The expression of CRBN in these samples was analyzed by immunohistochemistry (IHC). Scale bar: 50 μm. **b** Psa-AR binds to the PSMA protein to form a self-assembled structure. **c** MST ligand binding measurements between the Psa-AR and PSMA proteins, *n* = 3 per group. **d** The critical assembly concentration (CAC) of Psa-AR and Psa-AR supplemented with PSMA was determined via a standard ThT assay; *n* = 3 per group. **e** Kinetic self-assembly curves of Psa-AR (40 μM) and Psa-AR (40 μM) with PSMA added via the standard ThT assay; *n* = 3 per group. **f** FTIR spectra of Psa-AR (40 μM) and Psa-AR (40 μM) with added PSMA. **g** Representative TEM images of Psa-AR (40 μM) after the addition of PSMA for 0.5 h. **h** Representative TEM images of Psa-AR (40 μM) after the addition of PSMA for 1 h, 4 h and 8 h

AR and HSP90 dual-target Nano-PROTAC, abbreviated as Psa-AR, was engineered as a modular system comprising four functional domains: (1) the PSMA-targeting ligand PSMA-617 was engineered to selectively bind prostate cancer cells by leveraging PSMA, a molecular target exhibiting >100-fold overexpression in malignant versus normal prostate tissues^40^; (2) the self-assembly domain YYQNYQ drove supramolecular aggregation^41^; (3) the enzalutamide-derived AR-binding moiety^42^; and (4) the CRBN-recruiting E3 ligase ligand.^43^ Clinical validation of CRBN expression was performed via immunohistochemical (IHC) analysis of six prostate cancer samples, revealing high CRBN expression in 83.3% of the tumor cells (Fig. 1a), confirming its selection for targeted protein degradation. The chemical structure of Psa-AR was confirmed via high-performance liquid chromatography (HPLC) and matrix-assisted laser desorption/ionization time-of-flight mass spectrometry (MALDI-TOF-MS) (Supplementary Fig. 1). The enhanced selective uptake of Psa-AR is mechanistically governed by its target recognition-driven assembly, whereas the efficient degradation of AR/HSP90 complexes relies on fibril-like dynamic structural reorganization of the assembled nanostructure.

To validate the contribution of the self-assembly module in enhancing selective cellular uptake and achieving protein complex degradation, P-AR was engineered as a control. P-AR maintains identical PSMA-targeting module and degradation (enzalutamide-CRBN) motifs as Psa-AR but is structurally devoid of the self-assembly domain, thereby enabling direct evaluation of the effects of nanostructures on biological efficacy. The chemical structure was confirmed by HPLC and MALDI-TOF-MS (Supplementary Fig. 2). To elucidate these critical processes, we systematically characterized the solution-phase behavior of Psa-AR, focusing on its target-specific assembly kinetics and conformational adaptability under physio-mimetic conditions (Fig. 1b). First, microscale thermophoresis (MST) assays demonstrated strong binding affinity between Psa-AR and PSMA (Kd = 7.2 nM, Fig. 1c). The PSMA-bound conformation of Psa-AR is characterized by low energy and high stability, which lowers the critical assembly concentration (CAC) by 8.76-fold (95.5–10.9 μM), as revealed by our previous work, which revealed accelerated nucleation through stabilized π‒π stacking and hydrogen bonding interactions^44^ (Fig. 1d). In contrast, P-AR showed no detectable CAC upon PSMA addition within the tested concentration range (50 μM), confirming that the assembly motif triggered self-assembly behavior (Supplementary Fig. 3). Through assembly kinetics analysis, the PSMA-bound system achieved 50% assembly (t_1/2_) within 1.4 h, whereas the unbound Psa-AR system required 11.4 h (Fig. 1e). Full assembly equilibrium was attained at 12 h and 60 h, suggesting that PSMA binding stabilizes intermediate conformations to bypass high-energy nucleation barriers. Psa-AR assemblies with secondary structural transitions were probed via Fourier transform infrared (FTIR) spectroscopy. Deconvoluted amide I band analysis revealed distinct β-sheet-rich assemblies (peak at 1634 cm⁻¹) upon PSMA binding. In contrast, the unbound system exhibited random coil-dominated spectral signatures (1661 cm⁻¹ broad peak) with no obvious ordered secondary structures, confirming that PSMA engagement drives conformational ordering through β-sheet-mediated molecular packing (Fig. 1f). FTIR structural characterization revealed that P-AR maintains a disordered conformation even upon PSMA binding, with an absorption peak observed at 1635 cm⁻¹ due to the absence of the assembly domain (Supplementary Fig. 4). On this basis, we systematically investigated the structural evolution of Psa-AR assemblies upon PSMA binding via time-resolved transmission electron microscopy (TEM). TEM analysis revealed time-dependent morphological transformation. Within 0.5 h of PSMA engagement, spherical nanoparticles (200–250 nm diameter) dominated the assembly landscape (Fig. 1g). By 1 h, transitional architectures emerged where spherical assemblies coexisted with nascent nanofibers, ultimately maturing into well-defined fibrous networks after 8 h of incubation (Fig. 1h). No obvious nanostructures were detected in the P-AR group at 40 μM (Supplementary Fig. 5). This dynamic assembly mechanism, initiated by PSMA binding, enables programmable nanostructure evolution optimized for cell uptake and AR/HSP90 complex degradation.

### Nano-PROTAC design and functional validation

**Fig. 2:**
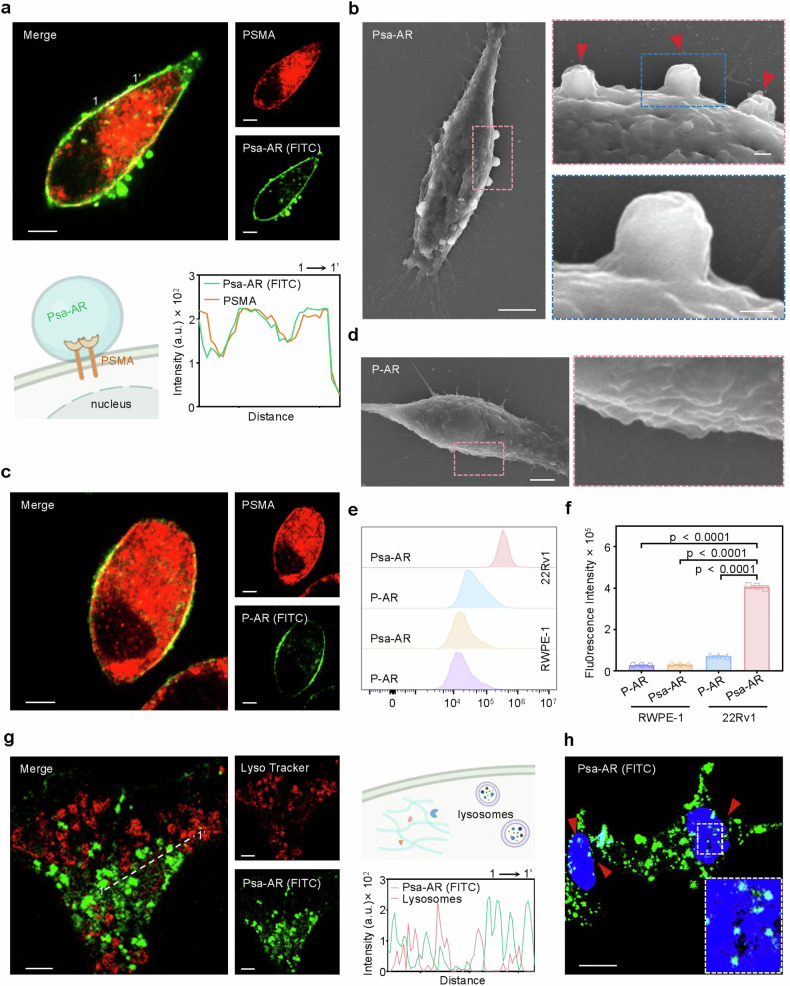
**a** Representative 22Rv1 cell CLSM images of FITC-labeled Psa-AR (40 μM) assembly (green channel) interacting with PSMA (red channel) and its colocalization analysis (1–1’). Scale bar: 10 μm. **b** Representative SEM images of 22Rv1 cells after treatment with Psa-AR (40 μM) for 4 h. Scale bar: 1 μm. Scale bar: 200 nm (zoom in). **c** Representative 22Rv1 cell CLSM images of FITC-labeled P-AR (40 μM) (green channel) interacting with PSMA (red channel). Scale bar: 10 μm. **d** Representative SEM images of 22Rv1 cells after treatment with P-AR (40 μM) for 4 h. Scale bar: 1 μm. Scale bar: 200 nm (zoom in). **e** The uptake of either Psa-AR or P-AR by 22Rv1 (PSMA^high^) and RWPE-1 (PSMA^low^) cells was examined by flow cytometry, followed by comprehensive quantitative analysis (**f**), *n* = 3 per group. **g** Representative 22Rv1 cell CLSM images of FITC-labeled Psa-AR assembly (green channel) with lysosomes (red channel) and colocalization analysis. Scale bar: 10 μm. **h** Representative CLSM images of 22Rv1 cells after incubation with FITC-labeled Psa-AR (green channel) for 12 h. Scale bar: 10 μm; blue channel: DAPI

Building upon the mechanistic basis of PSMA-triggered assembly and nanostructural transformation, we systematically evaluated the cellular-level selectivity, membrane assembly, and subcellular distribution of Psa-AR. First, confocal laser scanning microscopy (CLSM) demonstrated the predominant membrane localization of Psa-AR in PSMA^high^ 22Rv1 cells, which highly colocalized with PSMA (Fig. 2a). In PSMA^low^ PC3 cells, no obvious signal was observed after treatment with Psa-AR (Supplementary Fig. 6). Second, scanning electron microscopy (SEM) further revealed that PSMA triggered self-assembly, with discrete spherical assemblies observed on the 22Rv1 cell membrane within 30 min (Fig. 2b). Moreover, to spatiotemporally track the membrane-confined assembly, we engineered an NBD (nitrobenzoxadiazole)-labeled Psa-AR.^45^ This environment-sensitive fluorophore exhibits assembly enhanced fluorescence: monomeric probes remain quenched owing to intramolecular charge transfer, whereas PSMA-triggered supramolecular assembly sequesters NBD moieties into hydrophobic nanodomains, restoring fluorescence (Supplementary Fig. 7a). To elucidate the structural determinants of membrane-localized assembly, we performed comparative studies with the nonassembling P-AR control. CLSM analysis revealed significantly reduced membrane binding of P-AR relative to Psa-AR (Fig. 2c), whereas SEM imaging revealed the complete absence of membrane-associated nanostructures (Fig. 2d). These observations, combined with the minimal fluorescence enhancement of NBD-labeled P-AR at the membrane (Supplementary Fig. 7b), provide compelling evidence for an assembly unit-dependent self-assembly mechanism. Building on our previous findings demonstrating micropinocytosis-mediated cellular internalization, we systematically evaluated the selective uptake of FITC-labeled Psa-AR versus nonassembling P-AR in PSMA^high^ and PSMA^low^ cell lines via quantitative flow cytometry (Fig. 2e). Strikingly, compared with its nonassembling counterpart, Psa-AR exhibited a 5-fold greater uptake efficiency in 22Rv1 cells, accompanied by a 13-fold greater selectivity ratio between PSMA^low^ RWPE-1 and PSMA^high^ 22Rv1 cells (Fig. 2f). The selective self-assembly and uptake of Psa-AR and P-AR were further confirmed by IF, which revealed low cellular uptake of both Psa-AR and P-AR in RWPE-1 cells (Supplementary Fig. 8). To traffic the intracellular Psa-AR, we performed CLSM-based lysosomal colocalization analysis, which revealed minimal overlap with lysosomal compartments, indicating predominant cytoplasmic localization (Fig. 2g). Furthermore, nuclear staining demonstrated substantial nuclear accumulation potential of the compound. These spatial distribution characteristics provide a structural rationale for the capacity of Psa-AR to degrade AR proteins residing in both the cytoplasmic and nuclear compartments (Fig. 2h).

### Nano-PROTAC-mediated protein interaction and degradation

**Fig. 3:**
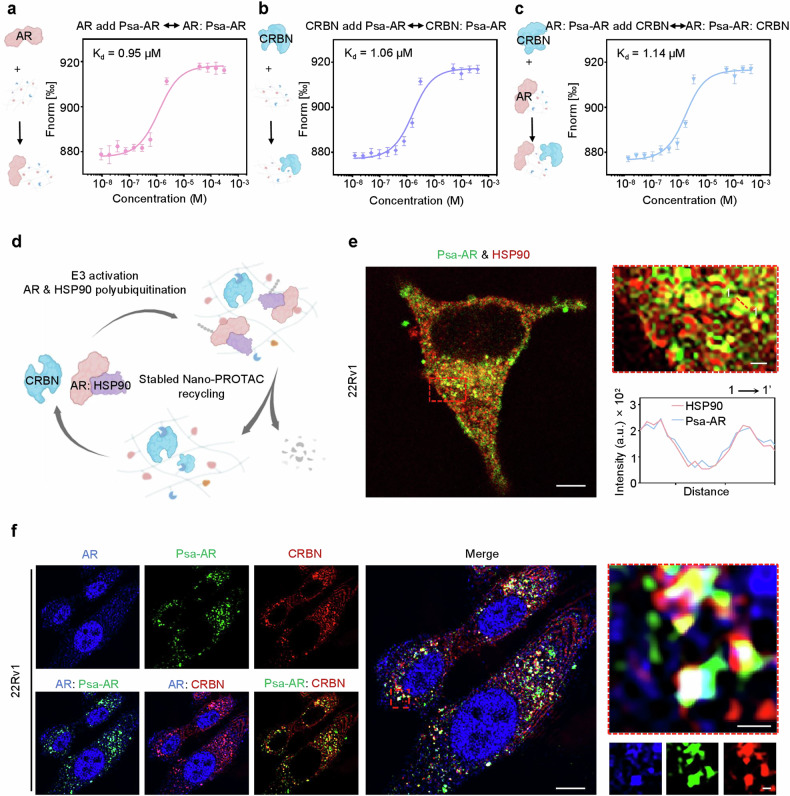
Polynary nanocomposite (AR: Psa-AR: CRBN) formation. **a** Dose‒response curve for the binding interaction between Psa-AR and the AR protein; *n* = 3 per group. **b** Dose‒response curve for the binding interaction between Psa-AR and the CRBN protein; *n* = 3 per group. **c** Dose‒response curve for the binding interaction between the binary complex (Psa-AR: AR) and CRBN, *n* = 3 per group. **d** In vitro Psa-AR self-assembly, protein binding and degradation. **e** Representative 22Rv1 cell CLSM images of FITC-labeled Psa-AR assembly (green channel) interacting with HSP90 (red channel) and its colocalization analysis (1–1’). Scale bar: 10 μm. **f** Colocalization of the ternary complex was observed in 22Rv1 cells via confocal microscopy; blue channel: AR; green channel: Psa-AR; red channel: CRBN; and colocalization image. Scale bar: 10 μm. Scale bar: 1 μm (zoom in)

**Fig. 4:**
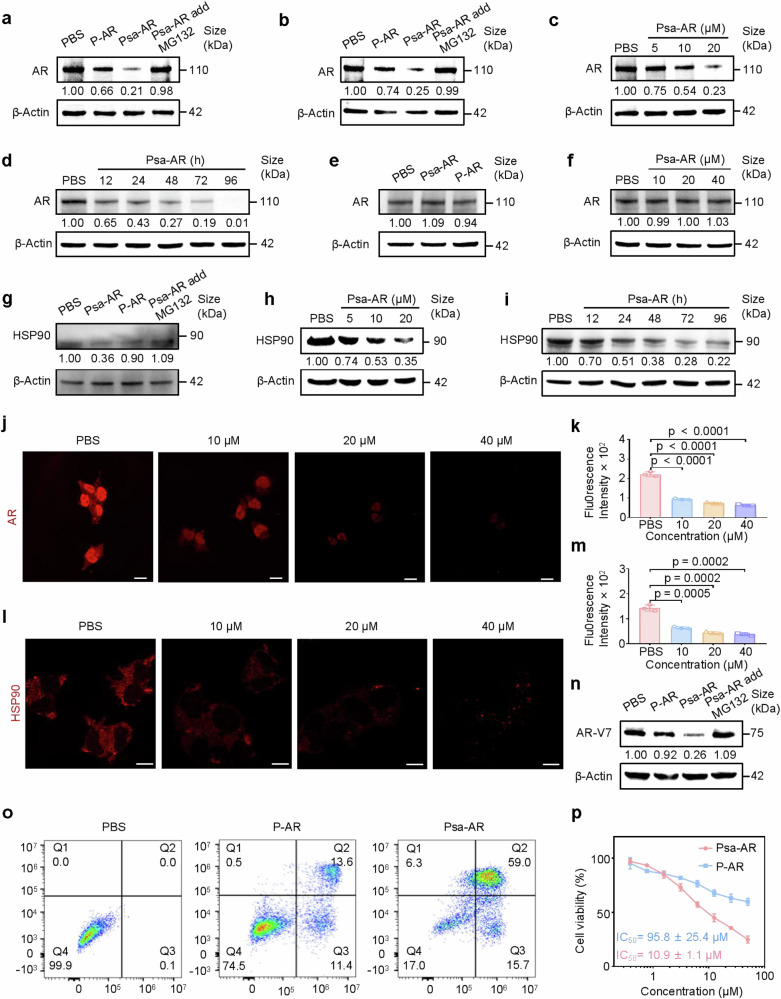
**a**, **b** AR expression in 22Rv1 cells and LNCaP cells after treatment with PBS, P-AR (20 μM), Psa-AR (20 μM) or Psa-AR (20 μM) combined with MG132 (2 μM) for 48 h, as determined by western blotting. β-Actin was used as an internal reference. **c** Western blot analysis of AR protein expression in 22Rv1 cells treated with Psa-AR (5 μM, 10 μM, or 20 μM) as indicated for 48 h. **d** Western blot analysis of AR protein expression in 22Rv1 cells treated with Psa-AR (12 h, 24 h, 48 h, 72 h or 96 h) as indicated at 20 μM. **e** AR expression in 22Rv1 (PSMA^KD^) cells after treatment with PBS, P-AR (20 μM) or Psa-AR (20 μM) for 48 h, as determined by western blotting. β-Actin was used as an internal reference. **f** Western blot analysis of AR protein expression in 22Rv1 (PSMA^KD^) cells treated with Psa-AR (10 μM, 20 μM, or 40 μM) as indicated for 48 h. **g** HSP90 expression in 22Rv1 cells after treatment with PBS, P-AR (20 μM), Psa-AR (20 μM) or Psa-AR (20 μM) combined with MG132 (2 μM) for 48 h, as determined by western blotting. β-Actin was used as an internal reference. **h**, **i** Evaluation of HSP90 expression in 22Rv1 cells under different conditions by immunoblotting. All uncropped blots are presented in Supplementary Fig. 26. **j**, **k** Representative 22Rv1 cell CLSM images of the degradation of AR after incubation with FITC-labeled Psa-AR for 12 h and quantitative analysis, *n* = 3 per group. Scale bar: 10 μm. **l**, **m** Representative 22Rv1 cell CLSM images of the degradation of HSP90 after incubation with FITC-labeled Psa-AR for 12 h and quantitative analysis, *n* = 3 per group. Scale bar: 10 μm. **n** AR-V7 expression in 22Rv1 cells after treatment with PBS, P-AR (20 μM), Psa-AR (20 μM) or Psa-AR (20 μM) combined with MG132 (2 μM) for 48 h, as determined by western blotting. β-Actin was used as an internal reference. **o** Results of apoptosis in 22Rv1 cells treated with PBS, P-AR (20 μM), or Psa-AR (20 μM) for 48 h, as detected by flow cytometry. X-axis: fluorescence signal of Annexin V, Y-axis: fluorescence signal of PI The data were analyzed via FlowJo software; *n* = 3 per group. **p** Cell viability evaluation of Psa-AR and P-AR in response to 22Rv1, *n* = 6 per group

The bifunctional ability of PROTACs to simultaneously engage both target proteins and E3 ligases, thereby mediating their proximity, constitutes the molecular foundation for efficient protein degradation. To characterize the ternary complex formation potential of Psa-AR, we conducted systematic assessments of its binding ability with AR and CRBN across both the solution phase and the cellular level. MST affinity assays revealed a binding affinity of 0.95 μM between Psa-AR and AR (Fig. 3a) but a comparable affinity (1.06 μM) for CRBN (Fig. 3b). Notably, in the presence of prebound AR, Psa-AR maintained an affinity of 1.14 μM for CRBN (Fig. 3c). These quantitative binding profiles collectively validate the heterobifunctional capacity of Psa-AR to coengage AR and CRBN within a physiologically relevant affinity range. Under physiological conditions, AR forms an inactive complex with the molecular chaperone HSP90 in the cytoplasm.^14^ HSP90 directly binds to the ligand-binding domain of AR, facilitating its proper folding and maintaining its conformational stability (Fig. 3d). The fibrillar network architecture formed by Psa-AR in the cytoplasm serves as a critical structural scaffold for the concurrent engagement of AR and HSP90 complexes. The IF results indicate that HSP90 can be enriched in the vicinity of Psa-AR, suggesting the possibility for the degradation of HSP90 (Fig. 3e). Using AR as a paradigm, multiplexed IF analysis demonstrated Psa-AR’s ability to colocalize AR, CRBN, and self-assembled nanostructures, indicative of productive ternary complex formation. Leveraging the proximity effect enabled by this fibrillar framework, CRBN anchored on the network simultaneously mediates ubiquitination tagging of both AR and HSP90, thereby establishing a supramolecular platform for coordinated polyubiquitination and subsequent degradation of compartmentalized oncoproteins^30,33,46^ (Fig. 3f). In contrast, no ternary complex was observed in PC3 cells (PSMA^low^, AR^low^) (Supplementary Fig. 9). Building upon these findings, we systematically evaluated the protein degradation efficacy of Psa-AR versus conventional P-AR. Initial western blot (WB) analysis of PSMA^high^ 22Rv1 cells demonstrated that, compared with PBS controls, Psa-AR achieved 79% AR degradation, representing a 45% enhancement over P-AR. MG-132 is a proteasome inhibitor that mimics the C-terminal structure of proteasomal substrate proteins^.^^47–49^ A proteasome-dependent mechanism was confirmed through MG-132 pretreatment, which abolished >80% of the degradation activity (Fig. 4a). Furthermore, in LNCaP prostate cancer models, Psa-AR exhibited a 49% superior degradation efficiency relative to P-AR (Fig. 4b), confirming the therapeutic advantages conferred by nanoassembly driven PROTAC engineering. We further systematically evaluated the dose- and time-dependent degradation efficacy of Psa-AR in 22Rv1 cells. The compound demonstrated concentration-dependent AR protein degradation within the 10–40 μM range, achieving 80% target depletion at 40 μM (Fig. 4c). Notably, Psa-AR exhibited a prolonged degradation effect, maintaining over 99% protein knockdown within the 96-h observation period, which was primarily promoted by the assembly induced retention effect produced by the in situ assembled nanostructures^50^ (Fig. 4d). To assess the selective degradation of Psa-AR, we constructed PSMA-knockdown (PSMA^KD^) 22Rv1 cells via siRNA-mediated knockdown (Supplementary Fig. 10). We conducted degradation assays in PSMA^KD^ 22Rv1 cells. Both Psa-AR and P-AR failed to induce significant protein degradation in 22Rv1 (PSMA^KD^) cells (Fig. 4e). Furthermore, even with increasing concentrations, Psa-AR maintained minimal off-target effects on AR degradation, collectively demonstrating its superior target specificity through ligand-directed precision (Fig. 4f). We next evaluated the HSP90-targeting degradation capacity of Psa-AR. Compared with the control compound P-AR, which did not significantly deplete HSP90, Psa-AR triggered >60% ubiquitination-mediated proteasomal degradation of HSP90 (Fig. 4g). The degradation exhibited both time- and concentration-dependent characteristics, achieving sustained 78% protein clearance over 96 h at optimal concentrations (Fig. 4h, i). The time-dependent AR and HSP90 degradation was further evaluated in LNCaP cells, with a 97% degradation rate for AR and a 70% degradation rate for HSP90 observed. (Supplementary Fig. 11). The DC50 was calculated for AR and HSP90 in both 22Rv1 and LNCaP cells. To measure AR protein expression levels after treatment with Psa-AR at concentrations ranging from 1 to 100 μM, the DC₅₀ values for AR protein degradation were determined to be 9.45 μM in the 22Rv1 cell line and 11.39 μM in the LNCaP cell line (Supplementary Fig. 12). To further validate the degradation effects on AR and HSP90, we performed IF-based quantification of AR and HSP90 levels, which confirmed the temporally progressive and dose-responsive degradation profiles of these compounds (Fig. 4j–m). In contrast, the degradation effect of P-AR on HSP90 and AR was also evaluated by IF, which was in agreement with the WB results (Supplementary Figs. 13, 14). Furthermore, we investigated the therapeutic potential of Psa-AR against the clinically relevant AR-V7 splice variant. The compound demonstrated enhanced degradation efficacy, achieving 66% greater AR-V7 degradation than did P-AR (Fig. 4n). Mechanistically, this enhanced potency may stem from concomitant HSP90 inhibition destabilizing the chaperone‒client axis, thereby promoting the ubiquitination-dependent clearance of AR-V7.^14^ These findings provide a mechanistic foundation for overcoming CRPC driven by therapy-resistant AR variants. To investigate the observed dual degradation of AR, AR-V7 and HSP90 at the cellular level, we comprehensively evaluated the downstream pharmacodynamic outcomes and cytotoxic efficacy. Psa-AR demonstrated superior apoptosis-inducing capacity compared with that of the PBS controls and P-AR, with Annexin V/PI costaining revealing 75% total apoptotic cells (early and late phases) versus 25% in the P-AR-treated group (Fig. 4o, Supplementary Fig. 15). The apoptotic effect was further confirmed by evaluation of the expression of caspase 3, which is an apoptosis marker, with obvious cleaved caspase-3 expression observed after Psa-AR treatment (Supplementary Fig. 16). This amplified apoptotic cascade translated to potent cell death, as evidenced by dose‒response profiling, which revealed an 11.6-fold lower IC50 for Psa-AR (10.9 ± 1.1 μM) than for P-AR (95.8 ± 25.4 μM) in 22Rv1 cells (Fig. 4p). The cellular cytotoxicity of Psa-AR (11.1 ± 1.4 μM) compared with that of P-AR (112.1 ± 25.6 μM) was further evaluated in LNCaP cells, with a similar increase in cytotoxicity (Supplementary Fig. 17). Additionally, Psa-AR exhibited negligible cytotoxicity in PSMA^low^ normal RWPE-1 cells across the tested concentrations (1–100 μM, 48 h of exposure), with cell viability maintained above 90% relative to that of the vehicle controls (Supplementary Fig. 18). This target-selective biosafety profile strongly correlates with the PSMA-dependent cellular internalization mechanism, demonstrating favorable safety profiles associated with ligand-guided precision while effectively circumventing off-target toxicity in nonmalignant tissues.

### Nano-PROTAC biodistribution evaluation

**Fig. 5:**
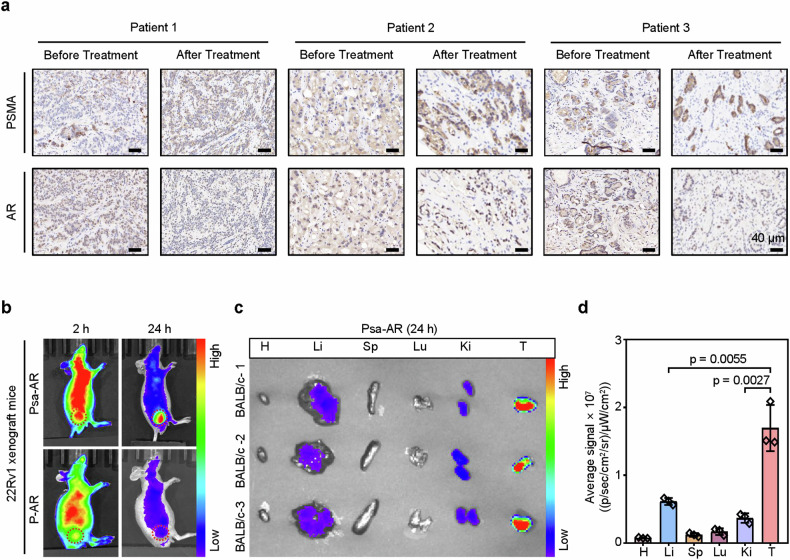
In vivo evaluation of Psa-AR enrichment and retention. **a** Representative immunohistochemical (IHC) staining of PSMA and AR expression in tumor tissues from three patients with prostate cancer before and after androgen deprivation therapy. The expression levels of PSMA and AR were assessed in serial sections at identical locations. Scale bars: 40 μm. **b** In vivo fluorescence images of 22Rv1 xenograft mice after treatment with Cy7-labeled P-AR and Psa-AR (30 mg/kg in 200 μL of PBS) for 2 h and 24 h. **c** Biodistribution in the tumors and main organs of 22Rv1 xenograft mice after injection with Cy7-labeled Psa-AR (30 mg/kg in 200 μL of PBS) for 24 h. (H Heart, Li Liver, Sp Spleen, Lu Lung, Ki Kidney, T Tumor). **d** Quantitative analysis of the fluorescence images in (**c**), *n* = 3 per group

Clinical longitudinal studies in CRPC patients have established an inverse correlation between AR downregulation and PSMA upregulation following targeted therapy. On the basis of these findings, we conducted a retrospective analysis of paired biopsy samples from three treatment-naïve CRPC patients and three posttherapeutic CRPC patients (*n* = 3) (Fig. 5a, Supplementary Fig. 19). IHC quantification revealed that AR suppression was accompanied by marked PSMA overexpression. This therapy-induced PSMA overexpression establishes a self-amplifying targeting cycle—the mechanism that potentiates Psa-AR’s selective tumor accumulation through PSMA-mediated endocytosis, creating a positive feedback loop between target engagement and drug delivery efficiency. To evaluate this mechanism in vivo, we systematically compared the biodistribution of Psa-AR and P-AR in tumor-bearing murine models (Fig. 5b). Following intravenous administration of Cy7-labeled conjugates (30 mg/kg), both compounds showed tumor-selective accumulation within 2 h, with Psa-AR resulting in 4-fold greater tumor deposition than P-AR. Notably, the nanostructured Psa-AR exhibited prolonged tumor retention, maintaining 78% of the residual signal at 24 h, in contrast to the rapid clearance of P-AR. This enhanced pharmacokinetic behavior was attributed to the self-assembled architecture-induced “assembly induced retention” (AIR) effect.^31^ On this basis, quantitative ex vivo biodistribution analysis (*n* = 3) at 24 h revealed superior tumor specificity of Psa-AR, with tumor-to-liver and tumor-to-kidney signal ratios of 2.8:1 and 4.6:1, respectively, which were greater than those of conventional PROTACs (Fig. 5c, d). The optimized biodistribution establishes Psa-AR as a therapeutically advantageous platform, simultaneously addressing targeted delivery challenges while minimizing systemic toxicity risks.

### In vivo protein degradation and antitumor efficiency of the nano-PROTAC

**Fig. 6:**
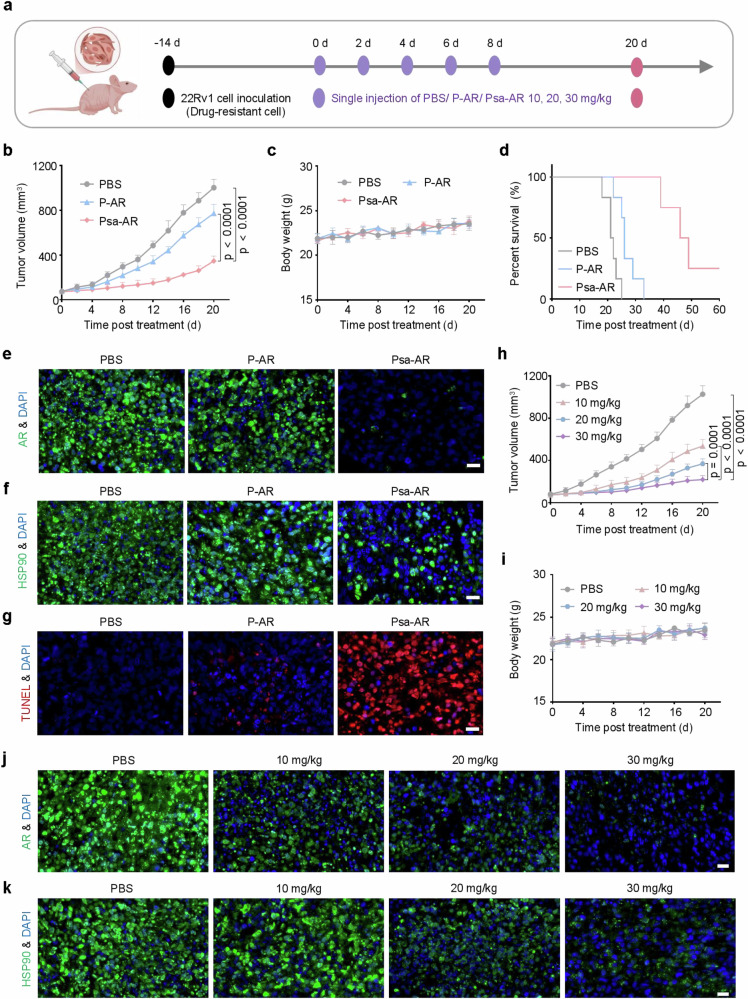
In vivo antitumor efficacy of Psa-AR. **a** Schematic illustration of the tumor inoculation and treatment protocol for 22Rv1 xenograft mice. (*n* = 6 per group) **b** Observation of the tumor inhibitory effect on 22Rv1 xenograft mice in PBS, P-AR, or Psa-AR (20 mg/kg in 200 μL of PBS) over 20 days of treatment. **c** Body weight changes in 22Rv1 xenograft mice in different treatment groups over 20 days. **d** Kaplan‒Meier survival curves of 22Rv1 xenograft mice in different groups over 20 days of treatment. **e** Immunofluorescence analysis of AR protein levels in 22Rv1 tumor tissues after treatment with PBS, P-AR, or Psa-AR (20 mg/kg in 200 μL of PBS) for 20 days. Scale bar: 20 μm. **f** Immunofluorescence analysis of the HSP90 protein levels in 22Rv1 tumor tissues after treatment with PBS, P-AR, or Psa-AR (20 mg/kg in 200 μL of PBS) for 20 days. Scale bar: 20 μm. **g** Terminal deoxynucleotidyl transferase (TdT) dUTP nick-end labeling (TUNEL) staining of tumors after treatment with PBS, P-AR, or Psa-AR (20 mg/kg in 200 μL of PBS) for 20 days. Scale bar: 20 μm. **h** Observation of the dose-dependent tumor inhibitory effect of 10 mg/kg, 20 mg/kg and 30 mg/kg Psa-AR on 22Rv1 xenograft mice over 20 days of treatment. **i** Changes in the body weights of 22Rv1 xenograft mice in different groups over 20 days of treatment. **j** Immunofluorescence analysis of AR protein levels in 22Rv1 tumor tissues after treatment with PBS or 10 mg/kg, 20 mg/kg or 30 mg/kg Psa-AR. Scale bar: 20 μm. *P* values were determined via one-way ANOVA followed by post hoc Tukey’s test. The data are presented as the means ± SDs. **k** Immunofluorescence analysis of the HSP90 protein levels in 22Rv1 tumor tissues after treatment with PBS or 10 mg/kg, 20 mg/kg or 30 mg/kg Psa-AR. Scale bar: 20 μm

**Fig. 7:**
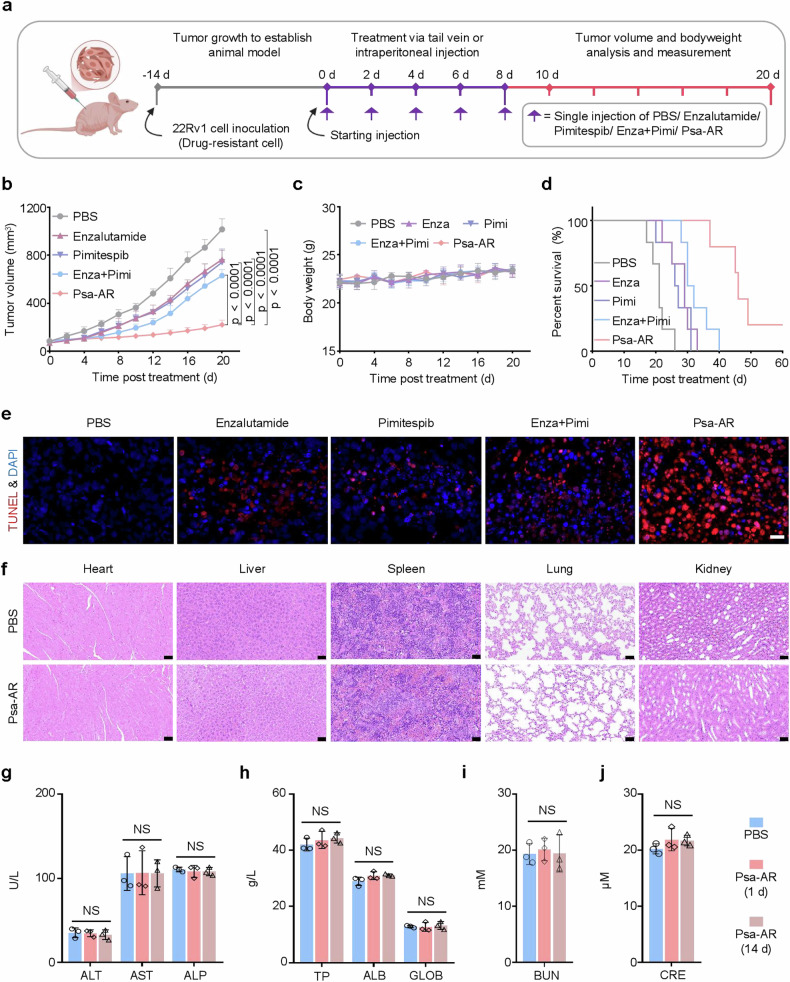
In vivo antitumor efficacy of Psa-AR. **a** Schematic illustration of the tumor inoculation and treatment protocol for 22Rv1 xenograft mice. (*n* = 6 per group) **b** Observation of the tumor inhibitory effect on 22Rv1 xenograft mice in PBS, enzalutamide, pimitespib, Enza, Pimi, and Psa-AR (30 mg/kg in 200 μL of PBS) over 20 days of treatment. (Enza, Pimi: Combination therapy with enzalutamide (AR antagonist) and Pimitespib (HSP90 inhibitor)). **c** Kaplan‒Meier survival curves of 22Rv1 xenograft mice in different groups over 20 days of treatment. **d** Changes in the body weights of 22Rv1 xenograft mice in different groups over 20 days of treatment. **e** Terminal deoxynucleotidyl transferase (TdT) dUTP nick-end labeling (TUNEL) staining of tumors after treatment with PBS, enzalutamide, pimitespib, ENZ, Pimi or Psa-AR (30 mg/kg in 200 μL of PBS) for 20 days. Scale bar: 50 μm. **f** Hematoxylin‒eosin (H&E) staining of the main organs after treatment with PBS or Psa-AR (30 mg/kg in 200 μL of PBS) for 24 h. Scale bar: 50 μm. **g**–**j** Blood biochemistry data, including ALT, AST, ALP, TP, ALB, GLOB, BUN, and CRE levels, of the mice after treatment with Psa-AR (20 mg/kg in 200 μL of PBS) for 1 day or 14 days (*n* = 3 per group). *p* values were determined via one-way ANOVA followed by post hoc Tukey’s test. The data are presented as the means ± SDs

To evaluate the therapeutic potential and superiority of Psa-AR in prostate cancer, we established subcutaneous xenograft tumor models using AR-mutant 22Rv1 cells.^51^ All procedures strictly complied with the Guide for the Care and Use of Laboratory Animals and were approved by the Committee for Animal Research of the National Center for Nanoscience and Technology. When the tumor volume reached ~100 mm³, 22Rv1-bearing mice were randomized into three groups (*n* = 6/group): the PBS control, Psa-AR, and P-AR groups. Treatments were administered via tail vein injection every other day for five cycles. The tumor volume and body weight were monitored every other day for 20 days (Fig. 6a). As a result, the Psa-AR-treated group reached 347 mm^3^, which was significantly lower than the P-AR (773 mm^3^) and PBS groups (1003 mm^3^). Psa-AR demonstrated tumor suppression efficacy, with 65% TGI, which exhibited a significant tumor suppression effect compared with P-AR (TGI: 23%) (Fig. 6b). The individual tumor growth curves for each group are presented in Supplementary Fig. 20. No obvious body weight loss was observed during treatment (Fig. 6c). K‒M survival analysis demonstrated significant therapeutic benefits conferred by Psa-AR, with treated cohorts exhibiting a median survival of 48 days, representing a 2.2-fold extension compared with that of PBS controls (22 days) and a 1.8-fold extension compared with that of P-AR controls (26 days) (Fig. 6d). To elucidate the mechanistic basis of tumor suppression, we quantitatively assessed AR and HSP90 protein expression in excised tumor tissues through IF. High-resolution spatial analysis revealed concerted proteolysis of both targets in the Psa-AR-treated group, resulting in AR and HSP90 reduction (Fig. 6e, f). This dual-target degradation profile directly correlates with the observed therapeutic efficacy, validating the assembly driven target engagement and degradation strategy. Histopathological validation via the terminal deoxynucleotidyl transferase (TdT) dUTP nick-end labeling (TUNEL) assay of tumor sections revealed a significant increase in the apoptotic index in Psa-AR-treated tumors compared with that in the PBS control and P-AR groups, which was the direct reason for tumor suppression (Fig. 6g). To address the critical limitations of conventional PROTACs, particularly the Hook effect-induced nonlinear pharmacokinetics and narrow therapeutic window. We rigorously evaluated the dose-responsive antitumor efficacy of Psa-AR in 22Rv1 xenograft models. Mice bearing subcutaneous tumors received escalating doses (10, 20, or 30 mg/kg) via intravenous administration every 48 h for five consecutive treatment cycles. The tumor volume and body weight were evaluated for 20 days. Tumor volume was suppressed in a dose-dependent manner, with a mean tumor volume of 1027 mm^3^ at 30 mg/kg, a mean tumor volume of 536 mm^3^ at 20 mg/kg and a mean tumor volume of 220 mm^3^ at 10 mg/kg (Fig. 6h). The individual tumor growth curves for each group are presented in Supplementary Fig. 21. There was no significant change in the body weight of the mice during the monitoring cycle, indicating that there was no significant toxicity at the administered dose (Fig. 6i). IF analysis of tumor sections provided spatially resolved dose-dependent quantification of AR and HSP90 proteolysis. High-resolution imaging revealed that progressive target degradation was correlated with increasing Psa-AR concentrations (Fig. 6j, k). Collectively, the dose-dependent tumor suppression and coordinated target proteolysis observed in the 22Rv1 xenograft model mechanistically validated the therapeutic superiority of in situ self-assembled Psa-AR. To systematically evaluate the therapeutic advantages of the dual-protein targeting strategy of Psa-AR through simultaneous degradation of AR and HSP90 in overcoming tumor drug resistance, this study compared the antitumor efficacy of Psa-AR with that of current clinical regimens (including the AR inhibitor enzalutamide, the HSP90 inhibitor pimitespib, and their combination) in 22Rv1 xenograft models (Fig. 7a). The experimental protocol was designed as follows: When the tumor volume reached ~100 mm³, the tumor-bearing mice were

Five groups (*n* = 6 per group) receiving PBS control, Psa-AR, enzalutamide monotherapy, pimitespib monotherapy, or enzalutamide‒pimitespib combination therapy were randomly assigned. All the treatments were administered via tail vein injection every other day for five cycles. Compared with the PBS control (1017 mm^3^), enzalutamide monotherapy (765 mm^3^), pimitespib monotherapy (742 mm^3^), and combination therapy (631 mm^3^) (Fig. 7b), the Psa-AR (220 mm^3^) significantly inhibited tumor growth in 22Rv1 model mice. Importantly, no significant body weight loss was observed in any group during the monitoring period, indicating favorable safety profiles (Fig. 7c). The individual tumor growth curves for each group are presented in Supplementary Fig. 22. Survival analysis further revealed that the Psa-AR group achieved a median survival of 46 days, which was significantly longer than that of the PBS control (21 days), enzalutamide (27 days), pimitespib (29 days), and combination groups (31 days) (Fig. 7d). To further elucidate the mechanism of action, TUNEL analysis of tumor tissue sections was performed via IF double-staining, which revealed increased activation of tumor apoptosis in the Psa-AR-treated group (Fig. 7e).

### In vivo biocompatibility evaluation of the nano-PROTAC degradation system

To thoroughly evaluate the in vivo biocompatibility of the Psa-AR degradation system, we performed comprehensive toxicological assessments in healthy BALB/c mice. At 24 h post-administration of either PBS or Psa-AR (30 mg/kg, *n* = 3 per group), we collected major organs (heart, liver, spleen, lungs, and kidneys) along with serum samples for histopathological and biochemical analysis. Hematoxylin and eosin (H&E)-stained tissue sections revealed preserved organ architecture in all treatment groups, with no observable pathological changes—including necrosis, inflammatory infiltration, or fibrosis—in any of the examined tissues (Fig. 7f). Complementary serum biochemistry profiling was used to evaluate the following hepatic and renal functional markers: alanine aminotransferase (ALT), aspartate aminotransferase (AST), alkaline phosphatase (AL), total protein (GLOB), albumin (ALB), blood urea nitrogen (BUN), and creatinine (CRE), which were not significantly different between the groups (Fig. 7g). The results of routine blood analysis, including white blood cell count (WBC), neutrophil (NEU), lymphocyte (LYM), red blood cell count (RBC), platelet count (PLT), hemoglobin (HGB) and mean platelet volume (MPV) levels, were further evaluated, and no obvious changes were detected (Supplementary Fig. 23). These comprehensive biosafety evaluations confirmed that the Psa-AR system maintains exceptional biocompatibility, with no acute toxicity observed in vital organs or systemic metabolic pathways. The combined histopathological and biochemical evidence strongly supports the clinical translatability of this targeted protein degradation platform.

## Discussion

CRPC remains a major therapeutic challenge largely due to resistance to AR pathway inhibitors such as enzalutamide and abiraterone. This resistance is primarily driven by persistent expression of AR splice variants (e.g., AR-V7) and the HSP90-mediated stabilization of oncoproteins that sustain tumor survival and progression. Conventional HSP90 inhibitors and PROTACs have shown limited clinical success, largely owing to systemic toxicity, suboptimal pharmacokinetic profiles, and insufficient degradation efficiency. These limitations highlight the urgent need for novel strategies that enhance targeted delivery and improve the therapeutic window.

Nanotechnology-based PROTAC delivery platforms have recently emerged as a promising solution to these challenges. Previous studies have demonstrated that nanostructured systems offer significant advantages in enhancing targeted delivery efficiency, reducing off-target toxicity, and promoting cooperative protein–protein interactions. Through surface functionalization, nanocarriers facilitate tissue-specific accumulation, thereby minimizing damage to healthy tissues. Moreover, their tunable surface properties provide an ideal foundation for multivalent ligand display. This multivalency not only strengthens the binding affinity between PROTAC molecules and target proteins but also promotes efficient formation of ternary complexes (POI: PROTAC: E3 ligase), substantially enhancing degradation efficacy.

Inspired by these advances, we developed a PSMA-targeted self-assembling nano-PROTAC (Psa-AR) for the simultaneous degradation of AR and HSP90. This innovative platform integrates three core functional elements: a PSMA-specific targeting ligand for tumor-selective accumulation, a self-assembling peptide backbone for controlled nanostructure formation, and dual protein-binding motifs capable of engaging both AR and HSP90 while recruiting the ubiquitin-proteasome machinery. The unique design enables proximity-induced ubiquitination and subsequent degradation of target proteins. In 22Rv1 CRPC models, Psa-AR induced 80% degradation of full-length AR, 74% degradation of AR-V7, and 65% depletion of HSP90, markedly outperforming existing PROTACs and small-molecule inhibitor combinations.

A critical analysis of these results underscores several fundamental advantages of our Psa-AR system. First, the PSMA-triggered self-assembly mechanism ensures that nanostructure formation occurs preferentially within the tumor microenvironment, enhancing local retention while reducing systemic exposure. This is corroborated by the 4-fold increase in tumor accumulation and prolonged intratumoral retention compared to the non-assembly control (P-AR). Second, the fibrillar architecture of the assembled nanostructures enables simultaneous engagement of AR and HSP90, leading to cooperative degradation that is unattainable with monovalent PROTACs. The notable degradation of AR-V7, a notoriously difficult therapeutic target, may be attributed to disrupted chaperone function following HSP90 depletion. Third, Psa-AR exhibits dose-dependent and sustained degradation kinetics.

Beyond immediate applications in CRPC, the modularity of this nano-PROTAC platform offers broad translational potential. Its design allows for the rational integration of alternative targeting ligands, protein-binding domains, and E3 ligase ligands, which can be further optimized through computational modeling and AI-driven predictive tools. The capability to spatially control protein degradation in vivo via proximity-induced effects provides a unique tool for probing complex protein interaction networks and signaling pathways. Moreover, by simply replacing the targeting moiety, this platform can be adapted to other malignancies, offering a generalized strategy for targeted protein degradation across a spectrum of cancers.

The development of Psa-AR represents a significant advancement in the field of targeted protein degradation, particularly in addressing the persistent challenge of therapeutic resistance in CRPC. Our results demonstrate that the integration of PSMA-mediated targeting with peptide self-assembly enables spatially controlled formation of fibrillar nanostructures within tumor cells, facilitating concurrent degradation of AR and HSP90. This dual-degradation approach is critical given the well-documented role of HSP90 in stabilizing AR and its splice variants, including AR-V7, which confer resistance to conventional AR-directed therapies. The observed 74% degradation of AR-V7 is particularly noteworthy, as this variant lacks the ligand-binding domain targeted by most AR inhibitors, making it notoriously difficult to inhibit through conventional means. By exploiting the chaperone function of HSP90, Psa-AR indirectly targets AR-V7 for proteasomal degradation, offering a promising strategy to overcome variant-driven resistance.

The modular design of Psa-AR provides a versatile platform that can be adapted to target other oncoproteins and cancer types. By replacing the PSMA-targeting moiety with ligands specific to other tumor-associated antigens (e.g., HER2, EGFR, or folate receptor), similar nano-PROTACs could be developed for other cancers. Additionally, the integration of computational modeling and AI-driven design tools could further optimize the binding affinity, specificity, and degradation efficiency of future constructs, enabling personalized therapeutic approaches based on individual tumor profiles. Moreover, the capability to spatially control protein degradation in vivo via proximity-induced effects provides a unique and powerful tool for probing complex protein interaction networks and signaling pathways, enabling to dissect context-specific functions of target proteins with high precision in living systems. This approach allows for the selective disruption of protein complexes in specific cellular compartments.

In conclusion, we have developed a novel and multifunctional nano-PROTAC system that leverages in vivo self-assembly to achieve concurrent degradation of AR and HSP90, effectively overcoming resistance mechanisms in CRPC. The platform demonstrates tumor specificity, durable degradation kinetics, and potent antitumor activity with minimal off-target effects. These findings not only present a new therapeutic strategy for CRPC but also establish a versatile and extensible framework for engineering next-generation nanotherapeutics capable of multiplexed oncoprotein targeting. We anticipate that this approach will inspire further innovations in targeted protein degradation and contribute to the development of more effective and safer treatments for advanced cancers.

## Materials and methods

### Study approval

All the animal experiments were performed in accordance with the Guide for Care and Use of Laboratory Animals, which was approved by the Committee for Animal Research of the National Center for Nanoscience and Technology, China (NCNST21-202503-0020). The use of human tissues for research was approved by the ethics committees of the National Cancer Center/National Clinical Research Center for Cancer/Cancer Hospital, Chinese Academy of Medical Sciences and Peking Union Medical College, China (22/115-3318).

### Binding affinity evaluated by microscale thermophoresis (MST)

MST (NanoTemper Technologies GmbH, Munich, Germany) was utilized to evaluate the binding affinity between Psa-AR and RED-NHS-labeled PSMA, AR, or CRBN proteins. The PSMA, AR, and CRBN proteins were labeled with the Protein Labeling Kit RED-NHS 2nd Generation (Amine Reactive). RED-PSMA, RED-AR, and RED-CRBN proteins were diluted to a final concentration of 400 nM with PBS-T buffer (0.05% Tween 20), where the fluorescence signals satisfied the typical detection limit of the Monolith NT.115 instrument (NanoTemper Technologies). The binding affinity between Psa-AR and the RED-NHS-labeled AR protein was assessed. For the binding affinity of the ternary complexes AR:Psa-AR and RED-CRBN, 10 µL AR protein and 10 µL Psa-AR were diluted 2:1 in 10 µL buffer to make 16-sample dilutions with final concentrations ranging from 1 mM to ca. 15 nM, respectively. Then, 10 µL of RED-CRBN was added to each sample to achieve a final concentration of 400 nM. After incubation for 20 min, the samples were loaded into standard/premium-treated capillaries. All the experiments were performed at 22 °C. The data were analyzed by NanoTemper analysis software.

### Cellular immunofluorescence by confocal laser scanning microscopy (CLSM)

22Rv1 cells were seeded at a density of 1 × 105 cells per well in glass-bottom culture dishes containing DMEM and incubated overnight. FITC-labeled Psa-AR (40 µM) was incubated with the cells for 12 h. Cellular imaging was performed via CLSM. Briefly, the cells were solidified with paraformaldehyde (4%) for 30 min. Then, a rabbit anti-PSMA monoclonal antibody and a rabbit anti-HSP90 monoclonal antibody were used to stain the PSMA and HSP90 proteins, respectively, to validate the binding of the assemblies to the PSMA and HSP90 proteins. A mouse anti-CRBN monoclonal antibody and a rabbit anti-AR monoclonal antibody were used to stain the CRBN and AR proteins to validate the binding of the assemblies to the CRBN and AR proteins. Finally, the cells were prepared for confocal laser scanning after being washed with PBS 3 times. The FITC signal was evaluated via a 488 nm laser, and the protein signal was evaluated via a 640 nm laser with a 40 × 40 oil immersion objective lens via CLSM. For the binding affinity of the ternary compounds, laser confocal scanning was performed via a 405 nm laser for CRBN protein signal detection, a 488 nm laser for FITC signal detection, and a 640 nm laser for AR protein signal detection, with a 40 × 40 oil immersion objective lens.

### Immunoblotting

The pretreated cells were resuspended in lysis buffer containing 50 mM Tris-HCl (pH 8.0), 150 mM NaCl, 1% (v/v) Triton X-100, and protease inhibitors. The protein concentration was determined via a BCA kit (Applygen). Each sample (50 g of protein) was subjected to SDS‒PAGE and then transferred to a PVDF membrane. The blots were blocked in 5% (wt/v) blocking buffer. After washing three times with TBST buffer (10 mM TBS solution containing 0.1% (v/v) Tween-20), the blots were incubated with primary antibodies at 4 °C on a shaker for 16 h. After washing with TBST buffer three times, the blot was incubated with secondary antibodies at room temperature on a shaker for 2 h. After being washed three times with TBST buffer, the blots were subsequently scanned on a Typhoon Trio Variable Mode Imager.

### Flow cytometry analysis

A total of 1 × 105 22Rv1 cells per well were seeded in 6-well plates in DMEM containing 10% fetal bovine serum and 1% penicillin‒streptomycin. After being cultured at 37 °C in a humidified atmosphere with 5% CO_2_ overnight, 22Rv1 cells were treated with Psa-AR or P-AR (40 µM) for 24 h. Then, the cells were collected to evaluate fluorescence expression via flow cytometry. Similarly, RWPE-1 cells were seeded at a density of 1 × 105 cells per well in 6-well K-SFM plates containing 10% fetal bovine serum and 1% penicillin‒streptomycin. After being cultured at 37 °C in a humidified atmosphere with 5% CO_2_ overnight, RWPE-1 cells were treated with Psa-AR or P-AR (40 µM) for 24 h. The cells were then collected and analyzed for fluorescence expression via flow cytometry.

### In vivo and ex vivo fluorescence imaging

BALB/c nude mice (6–8 weeks old, 16–18 g) were housed in a specific-pathogen-free (SPF) environment. All experiments were conducted in accordance with the *Guidelines for the Management and Use of Laboratory Animals* and were approved by the Animal Research Committee of the National Center for Nanoscience and Technology (NCNST 21-202503-0020). 22Rv1 xenograft mice were established by subcutaneously injecting 100 µL of 5 × 106 cells into the right flank of each mouse. When the tumor volume reached ~200 mm^3^, the mice were intravenously injected with Cy7-labeled Psa-AR (30 mg/kg in 200 µL of PBS) or P-AR (30 mg/kg in 200 µL of PBS). In vivo imaging was performed at 2 h and 24 h post-injection via an IVIS imaging system. For ex vivo fluorescence imaging, 22Rv1 xenograft mice were intravenously injected with Cy7-labeled Psa-AR (30 mg/kg in 200 µL of PBS) and sacrificed 24 h post-injection to excise major organs (heart, liver, spleen, lung, and kidney) and tumors.

### Antitumor efficacy of the Psa-AR degradation system

22Rv1 xenograft mice were established by subcutaneously injecting 100 µL (5 × 106) of cells into the right flank of BALB/c nude mice (6–8 weeks old, 16–18 g). After the tumor volume reached ~100 mm^3^, the mice were randomly separated into three, four or five groups (*n* = 6) at 14 days post-tumor inoculation. The mice were intravenously administered PBS, P-AR (20 mg/kg), or Psa-AR (10 mg/kg, 20 mg/kg, or 30 mg/kg) once every 2 days for a total of five times. Additionally, enzalutamide (25 mg/kg), pimitespib (15 mg/kg), or enzalutamide (25 mg/kg) + pimitespib (15 mg/kg) was administered orally once daily for a total of ten times. The body weight and tumor volume (V) were measured for analysis. The tumor volume (V) was calculated via the following formula: V = (longest diameter × shortest diameter^2^)/2.

## Supplementary information

Supplementary Material

## Data Availability

All data in the manuscript are available in the main text and its supplementary information. The relevant data are available at: 10.6084/m9.figshare.30164497.v1.
