# Supplementary material for: In vivo self-assembled nano-PROTAC for the dual degradation of AR and HSP90 to overcome castration-resistant prostate cancer resistance
Source: Signal Transduct Target Ther. 2025 Oct 15;10:346. doi: 10.1038/s41392-025-02444-z (PMC12521527; doi:10.1038/s41392-025-02444-z)
Supplement: Supplementary file 1 — Supplementary Material [file 41392_2025_2444_MOESM1_ESM.docx]

Supplementary Materials for

*In vivo* Self-assembled Nano-PROTAC for dual Degradation of AR and HSP90 to Overcome Castration-Resistant Prostate Cancer Resistance

Fei-Ya Yang ^a, c, d, #^, Ni-Yuan Zhang ^b, #^, Yang Yang ^b, g, #^, Dong Chen ^a, c, d^, Li-Yuan Wu ^f^, Wen-Kuan Wang ^a, c, d^, Hao-Xi Wang ^a, c, d^, Zhuan Wen ^b, g^, Ming-Ze Cai ^b^, Hao-Ze Li ^b, g^, Haojie Huang ^h^, Hong-Wei An ^b,*^, Hao Wang ^b, e,*^, Nian-Zeng Xing ^a, c, d, *^

Correspondence to: xingnianzeng@126.com (Nian-Zeng Xing)

**This PDF file includes:**

Materials and Methods

Figures. S1 to S26

**Materials and Methods**

**General information**

Except for special instructions, all organic reagents used in this study were purchased from commercially available sources. Fmoc amino acids were purchased from GL Biochem (Shanghai). Wang resins were purchased from KS-V PEPTIDE. Beta Actin Monoclonal antibody (66009-1-lg) was purchased from proteintech. Enzalutamide carboxylic acid, Thalidomide-NH-CH_2_-COOH and Enzalutamide (MDV3100) were purchased from Bidepharm (Shanghai). MG-132 (Z-Leu-Leu-Leu-al) and Pimitespib (TAS-116) were purchased from MedChemExpress (Monmouth Junction, NJ, USA). Anti-PSMA antibody [EP3253] (ab76104), Anti-Androgen Receptor (AR-V7 specific) antibody [EPR15656-290] (ab273500) and Anti-Hsp90 alpha antibody [68/Hsp90] (ab282108) were purchased from Abcam. Androgen Receptor (D6F11) XP Rabbit mAb^®^ #5153, CRBN (F4I7F) Rabbit mAb #60312 and HSP90 (C45G5) Rabbit mAb #4877 were purchased from Cell Signaling Technology. Androgen Receptor Polyclonal antibody (22089-1-AP) and PSMA/GCPII Polyclonal antibody (13163-1-AP) were purchased from proteintech. Penicillin and streptomycin (CK0008-100ML), and 4% paraformaldehyde (CK0014-500ML) were purchased from Beijing Chengzhi Kewei Biotechnology Co. Ltd. Cell Counting Kit-8 (AC11L054) was purchased from Shanghai Life-iLab Biotech Co., Ltd. 96-Well Plates (MBB-96-LD), Mycoplasma Removal Agent (MRA, M1056) and Hoechst 33342 (B2662) were purchased from LABLEAD Inc. (Beijing, China). Annexin V-FITC/PI Apoptosis Kit (E-CK-A211) was purchased from Elabscience Biotechnology Co.,Ltd. Goat Anti-Rabbit IgG/SAlexa Fluor 640 (K1034G-AF640) was purchased from (Bei jing Solarbio Science & Technology Co., Ltd.), Solarbio. CELLSAVING (C40100) was purchased from New Cell & Molecular Biotech. Human/Mouse/Rat Androgen R/NR3C4 Alexa Fluor® 488-conjugated Antibody (FAB5876G-100UG) was purchased from R&D Systems^TM^. ABflo® 488 Rabbit anti-Mouse CD24 mAb (A25470) was purchased from ABclonal Technology (WuHan, China). All the animal experiments were performed in accordance with the Guide for Care and Use of Laboratory Animals, which was approved by the Committee for Animal Research of National Center for Nanoscience and Technology, China (NCNST21-202503-0020). The subcutaneous tumor xenograft experiment endpoint was 15-20 mm at their largest dimension or maximum tumor volume of 2000 mm^3.

**Synthesis and Characterization of Psa-AR and P-AR**

Psa-AR and P-AR were synthesized using solid-phase peptide synthesis. The compounds were characterized by reversed-phase C-18 preparative high-performance liquid chromatography (HPLC) and matrix-assisted laser desorption/ionization time-of-flight mass spectrometry (MALDI-TOF-MS). The chemical structures, HPLC data, and mass spectra are provided in the supplementary information.

**Standard ThT Assay**

The Standard ThT Assay was carried out to evaluate the self-assembling kinetics of reaction and protein induced assembly. Psa-AR (40 µM) and ThT (20 µM) were co-incubated in the presence or absence of PSMA (40 µM). The time-dependent ThT fluorescence intensity at 490 nm was monitored under excitation wavelength at 450 nm. The fluorescence intensity was normalized to present the self-assembling kinetics process. The data points were fitted to the Finke-Watzky (F-W) two-step model.

**Thioflavin T (ThT) Assay for Determination of Critical Assembly Concentration (CAC)**

The critical assembly concentration (CAC) was determined by measuring ThT fluorescence (20 µM) at different concentrations of Psa-AR (ranging from 5 to 200 µM) and Psa-AR with PSMA in H_2_O/CH_3_CN (v/v = 99:1) using a microplate reader (λ_ex_ = 450 nm, λ_em_ = 490 nm).

**Infrared spectrum (IR).**

Psa-AR (40 μM), and Psa-AR (40 μM) with PSMA are respectively dissolved in H_2_O/CH_3_CN (v/v = 99: 1). Drop it on the calcium fluoride infrared window, and after it is naturally dried, measure the infrared spectrum of 4000 cm^-1^to 1000 cm^-1^ with Fourier infrared spectrometer.

**TEM Imaging**

The morphology of the superstructure was characterized by transmission electron microscopy (TEM). Psa-AR (40 µM) and P-AR (40 µM) was placed on carbon-coated copper grids for 20 minutes at room temperature. Psa-AR (40 µM) and PSMA protein were co-incubated for 0.5 h, 1 h 4 h and 8 h, respectively. The surface deposited aggregation was negatively stained with 2% uranyl acetate for 3 min before the TEM studies.

**Cell Culture**

The 22Rv1 and LNCaP cell lines were purchased from the Cell Culture Center of the Institute of Basic Medical Sciences, Chinese Academy of Medical Sciences (Beijing, China). The LNCaP cell line was cultured in RPMI 1640 medium, and the 22Rv1 cell line was cultured in DMEM medium supplemented with 12% fetal bovine serum, 100 U mL^−1^ penicillin, and 100 µg mL^−1^ streptomycin in a humidified incubator containing 5% CO_2_ (Thermo, Waltham, MA, USA).

**Cellular uptake evaluation of Psa-AR**

22Rv1 cells were seeded into confocal microscope culture dishes at a density of 1 × 10^5 cells per well. FITC-labeled Psa-AR was co-incubated with 22Rv1 cells for different time periods, followed by washing with PBS three times. The assembly process was monitored by confocal laser scanning microscopy (CLSM) using a 488 nm laser excitation. Hoechst 33342 (1 mg/mL; Life Technologies) was used to stain the nucleus of cells at 37°C for 10 min. Finally, the cells were prepared for confocal laser scanning after washing with PBS for three times. The nucleus signal was evaluated by 405-nm laser. The FITC fluorescence of Psa-AR was quantitatively analyzed using ImageJ software.

**Selective Assembly and Retention of Psa-AR**

The selective self-assembly process of Psa-AR in 22Rv1 cancer cells and normal cells was quantitatively revealed. Briefly, cells (2 × 10^6) were seeded into 6 cm dishes, treated with Psa-AR for 12 hours and washed three times with PBS. Then cells were digested with trypsin at different time points, collected and resuspended with 500 μL PBS. The fluorescence intensity was measured using an FS 5 fluorescence spectrophotometer (λex = 450 nm).

**DC_50_ by Flow Cytometry Analysis**

A density of 1 × 10^5 22Rv1 cells per well were seeded in the 6-well plates in DMEM medium contained 10% fetal bovine serum and 1% penicillin-streptomycin. After cultured at 37°C in a humidified atmosphere with 5% CO_2_ overnight, 22Rv1 cells were treated with Psa-AR and P-AR at different concentration for 48 hours, respectively. Add AR conjugated antibody or HSP90 conjugated antibody and incubate for 6 h, respectively. Then cells were collected to evaluate the fluorescence expression using flow cytometry. Similarly, LNCaP cells were seeded at a density of 1 × 10^5 cells per well in the 6-well plates in 1640 medium containing 10% fetal bovine serum and 1% penicillin-streptomycin. After cultured at 37°C in a humidified atmosphere with 5% CO_2_ overnight, LNCaP cells were treated with Psa-AR and P-AR at different concentrationfor 48 hours, respectively. Add conjugated antibody and incubate for 6 h. Cells were then collected and analyzed for fluorescence expression using flow cytometry.

**Cell Viability Assays**

Cell viability was assessed using the CCK-8 assay. Briefly, 22Rv1 cells were seeded at a density of 5 × 10^3 cells per well in a 96-well plate containing DMEM medium supplemented with 10% fetal bovine serum and 1% penicillin-streptomycin. For 22Rv1 cells, Psa-AR and P-AR were dispersed in DMEM medium at various concentrations (5, 10, 20, 40, 60, 80, 100 µM) and added to each well for 24 hours of incubation. For RWPE-1 cells, Psa-AR was dispersed in DMEM medium at various concentrations (5, 10, 20, 40, 60, 80, 100 µM) and added to each well for 24 hours of incubation. After removing the sample solution, 100 µL of CCK-8 solution was added to each well and incubated for an additional 2 hours. The UV-Vis absorptions of sample wells (Asample), Ablank and control wells (Acontrol) were obtained by measuring the test absorption at 450 nm and a reference wavelength at 690 nm utilizing a Microplate reader, respectively. The cell viability (%) was calculated by the following equation: (Asample - Ablank)/ (Acontrol - Ablank) × 100. All the experiments were repeated for three times.

**Tumor Slices and Staining**

22Rv1 tumor tissues were harvested and fixed with 4% paraformaldehyde solution after treatment with PBS, P-AR, and Psa-AR at a dose of 20 mg/kg. The immunofluorescence staining of AR and HSP90, as well as terminal deoxynucleotidyl transferase deoxyuridine triphosphate (dUTP) nick end labeling (TUNEL) assay procedure were performed by Wuhan Servicebio Technology Co., Ltd. Similarly, 22Rv1 tumor tissues were harvested and fixed with 4% paraformaldehyde solution after treatment with after treatment with PBS and three doses of Psa-AR (10 mg/kg, 20 mg/kg, and 30 mg/kg). The immunofluorescence staining for AR and HSP90 expression was performed by Wuhan Servicebio Technology Co., Ltd. Additionally, 22Rv1 tumor tissues were harvested and fixed with 4% paraformaldehyde solution after treatment with PBS, Enzalutamide (25 mg/kg), pimitespib (15 mg/kg), Enzalutamide (25 mg/kg) + pimitespib (15 mg/kg) and Psa-AR (30 mg/kg). The immunofluorescence staining for AR and HSP90 and TUNEL assays were performed by Wuhan Servicebio Technology Co., Ltd.

**Toxicology Evaluation of Psa-AR Degradation System**

Healthy Balb/c nude mice (6–8 weeks old, 16–18 g, n = 3) were treated with PBS and Psa-AR at a dose of 30 mg/kg for 7 day and 14 days. Subsequently, the mice were sacrificed to excise major organs (heart, liver, spleen, lung, and kidney) and collect serum. Hematoxylin and eosin (H&E) staining of major organs was performed by Wuhan Servicebio Technology Co., Ltd. for histological evaluation. Blood biochemical analysis, including alanine aminotransferase (ALT), aspartate aminotransferase (AST), alkaline phosphatase (ALP), total protein (TP), albumin (ALB), globulin (GLOB), blood urea nitrogen (BUN), and creatinine (CRE), was conducted using a Hitachi Automatic Biochemical Analyzer 7100.

**Figure. S1.**

**Supplementary Fig. 1.** Characterization of Psa-AR. (a) Chemical structure of Psa-AR. (b) HPLC analysis of Psa-AR. The method of HPLC spectra was as follows: solvent A, 0.1% trifluoroacetic acid in 100% water; solvent B, 0.1% trifluoroacetic acid in 100% acetonitrile; 0 min-25 min, 5%-90% solvent B. (c) MALDI-TOF analysis of Psa-AR.

**Figure. S2.**

**Supplementary Fig. 2.** Characterization of P-AR. (a) Chemical structure of P-AR. (b) HPLC analysis of P-AR. The method of HPLC spectra was as follows: solvent A, 0.1% trifluoroacetic acid in 100% water; solvent B, 0.1% trifluoroacetic acid in 100% acetonitrile; 0 min-25 min, 5%-90% solvent B. (c) MALDI-TOF analysis of P-AR.

**Figure. S3.**

**Supplementary Fig. 3.** The critical assembly concentration of P-AR by standard ThT assay.

**Figure. S4.**

**Supplementary Fig. 4.** FTIR spectra of P-AR in the 40 μM.

**Figure. S5.**

**Supplementary Fig. 5.** Representative TEM images of the P-AR (40 μM). Scale bar: 100 nm.

**Figure. S6.**

**Supplementary Fig. 6.** (a) CLSM images of PC-3 cells after treated with FITC labeled Psa-AR. Green channel: FITC labeled Psa-AR. Red channel: PSMA protein. Blue channel: DAPI. Scale bar: 20 μm. (b) CLSM images of PC-3 cells after treated with FITC labeled P-AR. Green channel: FITC labeled P-AR. Red channel: PSMA protein. Blue channel: DAPI. Scale bar: 20 μm.

**Figure. S7.**

**Supplementary Fig. 7.** (a) CLSM images of 22Rv1 cells after treatment with Psa-AR-NBD (40 μM, 30 min). Scale bar: 10 μm. (b) CLSM images of 22Rv1 cells after treatment with P-AR-NBD (40 μM, 30 min). Scale bar: 10 μm.

**Figure. S8.**

**Supplementary Fig. 8.** (a) Representative CLSM images of 22Rv1 cells after incubation with FITC labeled Psa-AR and P-AR (green channel) with 40 μM for 12 h, scale bar: 10 μm, blue channel: DAPI. (b) Representative CLSM images of RWPE-1 cells after incubation with FITC labeled Psa-AR and P-AR (green channel) with 40 μM for 12 h. Scale bar: 10 μm. Blue channel: DAPI.

**Figure. S9.**

**Supplementary Fig. 9.** The colocalization of the ternary complex was not observed in PC3 cells, blue channel: AR, green channel: Psa-AR, red channel: CRBN. Scale bar: 10 μm.

**Figure. S10.**

**Supplementary Fig. 10.** PSMA expression before and after knockout of PSMA protein in 22Rv1 cells by western blots. β-Actin was used as an internal reference.

**Figure. S11.**

**Supplementary Fig. 11.** (a) Western blot analysis of AR protein expression in LNCaP cells treated with Psa-AR (12 h, 24 h, 48 h, 72 h or 96 h) as indicated with 20 μM. β-Actin was used as an internal reference. (b) Western blot analysis of HSP90 protein expression in LNCaP cells treated with Psa-AR (12 h, 24 h, 48 h, 72 h or 96 h) as indicated with 20 μM. β-Actin was used as an internal reference.

**Figure. S12.**


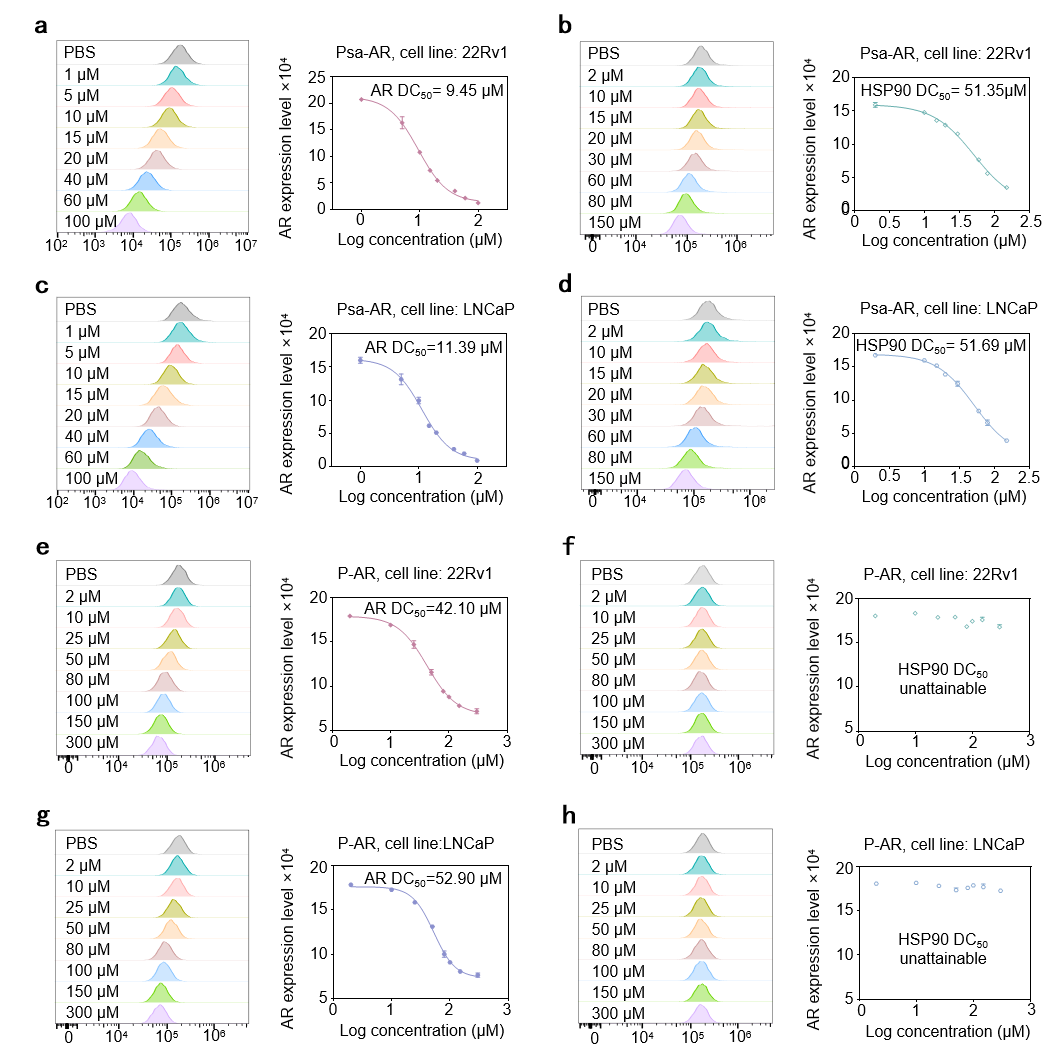


**Supplementary Fig. 12.** (a, b) Investigating the DC50 of Psa-AR that degrades the AR and HSP90 protein in cell 22Rv1 by flow cytometry. (c, d) Investigating the DC50 of Psa-AR that degrades the AR and HSP90 protein in cell LNCaP by flow cytometry. (e, f) Investigating the DC50 of P-AR that degrades the AR and HSP90 protein in cell 22Rv1 by flow cytometry. (g, h) Investigating the DC50 of P-AR that degrades the AR and HSP90 protein in cell LNCaP by flow cytometry.

.

**Figure. S13.**

**Supplementary Fig. 13.** Representative 22Rv1 cells CLSM images of the degradation of AR after incubation with FITC labeled P-AR (40 μM) for 12 h. Scale bar: 20 μm.

**Figure. S14.**

**Supplementary Fig. 14**. Representative 22Rv1 cells CLSM images of the degradation of HSP90 after incubation with FITC labeled P-AR (40 μM ) for 12 h. Scale bar: 20 μm.

**Figure. S15.**

**Supplementary Fig. 15.** Quantitative analysis of the results of flow cytometry for the detection of apoptosis in 22Rv1 cells after 48 h treatment with PBS, P-AR, Psa-AR (20 μM).

**Figure. S16.**

**Supplementary Fig. 16.** Cleaved Caspase-3 expressions in 22Rv1 cells after treatment with P-AR (20 μM) and Psa-AR (20 μM) for 48 h by western blots. β-Actin was used as an internal reference.

**Figure. S17.**

**Supplementary Fig. 17.** The cell viability evaluation of Psa-AR and P-AR to LNCaP.

**Figure. S18.**

**Supplementary Fig. 18.** The cell viability evaluation of Psa-AR to RWPE-1.

**Figure. S19.**


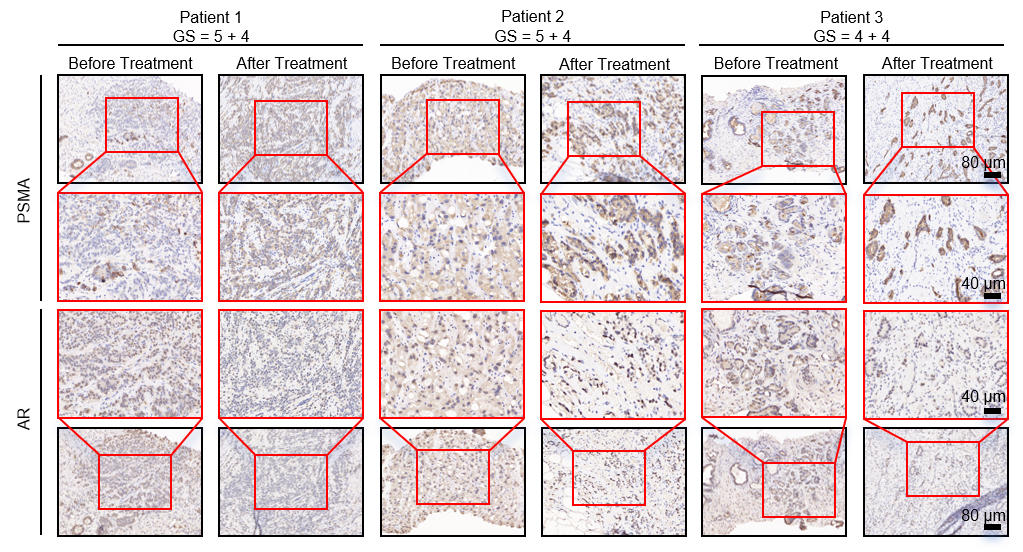


**Supplementary Fig. 19.** Representative immunohistochemical (IHC) staining of PSMA and AR expression in tumor tissues from three patients with prostate cancer before and after androgen deprivation therapy. Expression levels of PSMA and AR were assessed in serial sections at identical locations. Scale bars: 80 μm (low magnification). Scale bars: 40 μm (high magnification).

**Figure. S20.**

**Supplementary Fig. 20.** The individual tumor growth curves of each mouse in the PBS, P-AR and Psa-AR (20 mg/kg in 200 μL PBS) group.

**Figure. S21.**

**Supplementary Fig. 21.** The individual tumor growth curves of each mouse in the PBS, 10 mg/kg, 20 mg/kg and 30 mg/kg Psa-AR group.

**Figure. S22.**

**Supplementary Fig. 22.** The individual tumor growth curves of each mouse in the PBS, Enzalutamide (25 mg/kg), Pimitespib (15 mg/kg), Enza (25 mg/kg) + Pimi (15 mg/kg) and Psa-AR (30 mg/kg in 200 μL PBS) group. (Enza + Pimi: Combination treatment with Enzalutamide and Pimitespib).

**Figure. S23.**

**Supplementary Fig. 23.** Blood routine analysis including WBC, NEU, LYM, RBC, PLT, HGB and MPV levels of the mice after treatment with Psa-AR (20 mg/kg in 200 μL PBS) for 1 day and 14 days, respectively (n=3). p values were performed with one-way ANOVA followed by post hoc Tukey’s test.

**Figure. S24.**

**Supplementary Fig. 24. (a)** Original data for Flow cytometry in Fig. 2e. (b) Original data for Flow cytometry in Supplementary Fig. 12a-h.

**Figure. S25.**

**Supplementary Fig. 25.** Original data for Flow cytometry in Fig. 4o.

**Figure. S26.**

**Supplementary Fig. 26.** (a) The uncropped blots for Fig. 4a-i, n. (b) The uncropped blots for Supplementary Fig. 10, 11a, 11b, 13.
